# Statins modulate the expression profile of ATP-binding cassette transporters in human neuroblastoma cells

**DOI:** 10.1186/1471-2210-11-S2-A23

**Published:** 2011-09-05

**Authors:** Bihter Atil, Evelyn Sieczkowski, Martin Hohenegger

**Affiliations:** 1 Institute of Pharmacology, Center of Physiology and Pharmacology, Medical University of Vienna, 1090 Vienna, Austria

## Background

The development of chemoresistance still is a major problem in cancer therapy. Mainly, impact of chemotherapy is based on the upregulation of ATP-binding cassette (ABC) transporters, which correlates with bad prognosis and less chemotherapeutic success. Previously, we could show that the HMG-CoA reductase inhibitor simvastatin is able to inhibit the most prominent ABC transporter ABCB1. Additionally, we demonstrated that simvastatin coadministrated with the anthracycline doxorubicin led to increased apoptosis in neuroblastoma cells, and that these effects were comparable with the potential of verapamil, a first generation inhibitor of ABCB1 [1].

## Methods

The neuroblastoma cell line SH-SY5Y was used for our analyses. Alterations in the protein level of ABC transporters were demonstrated by Western blot analyses and in more detail with FACS. RNA from SH-SY5Y cells treated with simvastatin for 6 and 72 hours was isolated, and the mRNA levels of various ABC transporters were quantified by real-time PCR.

## Results

Simvastatin exposure led to a concentration-dependent decrease of ABCB1. Similarly, FACS analyses demonstrated a significant decrease of ABCB1 expression on the cell surface. Moreover, doxorubicin-induced elevations in ABCB1 cell surface expression were reversed by simvastatin. However, compensation of ABCB1 by other ABC transporters like ABCC1 and ABCC4 could not be detected. Conversely, ABCG2 showed upregulation on protein and mRNA level.

## Conclusions

Here we show that simvastatin is able to modulate ABCB1 expression. This potential seems to be divided in immediate and long-term effects. Expression of ABCB1 was downregulated on protein as well as on mRNA level after long-term incubation and partially compensated by other ABC transporters. Moreover, we also observed short-term regulation of the cell surface expression without any changes in total cellular level which might be based on impact of simvastatin in ABCB1 turn-over. Based on our findings, we suggest that simvastatin is a promising candidate as an adjuvant chemotherapeutic drug to impair transporter-mediated multidrug resistance.
